# WORKING THE NIGHT SHIFT CAUSES INCREASED VASCULAR STRESS AND DELAYED RECOVERY IN YOUNG WOMEN

**DOI:** 10.3109/07420528.2010.498067

**Published:** 2010-08-26

**Authors:** Shih-Hsiang Lo, Lian-Yu Lin, Jing-Shiang Hwang, Yu-Yin Chang, Chiau-Suong Liau, Jung-Der Wang

**Affiliations:** ^a^ Department of Internal Medicine, Zhongxing Branch of Taipei City Hospital, Taipei, Taiwan; ^b^ Institute of Occupational Medicine and Industrial Hygiene, College of Public Health, National Taiwan University, Taipei, Taiwan; ^c^ Department of Internal Medicine, National Taiwan University Hospital and College of Medicine, National Taiwan University, Taipei, Taiwan; ^d^ Institute of Statistical Science, Academia Sinica, Taipei, Taiwan; ^e^ Cardiovascular Center, Buddhist Tzu Chi General Hospital, Taipei Branch, Taipei, Taiwan; ^f^ Department of Environmental and Occupational Medicine, National Taiwan University Hospital, Taipei, Taiwan

**Keywords:** Ambulatory electrocardiographic monitoring, Blood pressure, Heart rate variability, Night shift, Shiftwork

## Abstract

Shiftwork has been associated with elevated blood pressure (BP) and decreased heart-rate variability (HRV), factors that may increase the long-term risk of cardiovascular-related mortality and morbidity. This study explored the effect of shiftwork on dynamic changes in autonomic control of HRV (cardiac stress), systolic BP and diastolic BP, i.e., SBP and DBP (vascular stress), and recovery in the same subjects working different shifts. By studying the same subjects, the authors could reduce the effect of possible contribution of between-subject variation from genetic predisposition and environmental factors. The authors recruited 16 young female nurses working rotating shifts—day (08:00–16:00 h), evening (16:00–00:00 h), and night (00:00–08:00 h)—and 6 others working the regular day shift. Each nurse received simultaneous and repeated 48-h ambulatory electrocardiography and BP monitoring during their work day and the following off-duty day. Using a linear mixed-effect model to adjust for day shift, the results of the repeated-measurements and self-comparisons found significant shift differences in vascular stress. While working the night shift, the nurses showed significant increases in vascular stress, with increased SBP of 9.7 mm Hg. The changes of SBP and DBP seemed to peak during waking time at the same time on the day off as they did on the working day. Whereas HRV profiles usually returned to baseline level after each shift, the SBP and DBP of night-shift workers did not completely return to baseline levels the following off-duty day (*p* < .001). The authors concluded that although the nurses may recover from cardiac stress the first day off following a night shift, they do not completely recover from increases in vascular stress on that day. (Author correspondence: jdwang@ntu.edu.tw)

## INTRODUCTION

Shiftwork has been associated with elevated blood pressure (BP) and decreased heart-rate variability (HRV), two factors that may increase the long-term risk of cardiovascular disease mortality and morbidity (Furlan et al., 2000; Harrington, 1994; Knutsson et al., 1986; Taylor & Pocock, 1972; Tenkanen et al., 1997; Vrijkotte et al., 2000). The interference of circadian rhythm by shiftwork may also increase psychosomatic, psychoneurotic, and gastrointestinal disorders (Axelsson et al., 2006; Moreno et al., 2006; Reinberg et al., 2007).

Both HRV and BP are controlled by autonomic nervous system activity, which oscillates over the 24 h (Quan et al., 1997). The mismatch and desynchronization between circadian rhythms and shiftwork may influence the fluctuations of autonomic activity, as shown in studies of plasma and urine catecholamines (Fujiwara et al., 1992; Rutenfranz et al., 1988; Yamasaki, 1998) and HRV monitoring (Freitas et al., 1997). However, the aforementioned studies have not taken into consideration the contribution of between-subject variation from genetic predisposition and environmental factors. Many studies of twins have found individual differences in HRV and BP variation to be influenced by genetic predisposition. Between 13% and 39% of twins are reported to have similar HRVs, but the percentage increases to 51% in times of stress. These studies suggest that people have their own basic patterns of HRV and BP (Boomsma et al., 1990; Busjahn et al., 1998; Degaute et al., 1994; Fava et al., 2005; Kupper et al., 2004; Singh et al., 1999; Sinnreich et al., 1999; Snieder et al., 1997).

In the present study, we performed simultaneous 48-h ambulatory electrocardiography (ECG) and BP monitoring of the same group of female nurses as they rotated through different shifts to explore the effects of shiftwork on the subjects' circadian patterns of HRV and BP and their recovery after different shifts. The repeated-measurements of these variables in the same subjects may help reduce contributions of between-subject variation and environment.

## METHODS

### Subjects

Subjects were recruited from a municipal hospital in Taipei, Taiwan, with 600 beds and 330 nurses. We recruited eight nurses from the Intensive Care Unit (ICU) and eight from the Internal Medicine Ward (IMW) who worked rotating shifts. Eight additional nurses from the same hospital's outpatient clinic (OPC), who worked only the regular day shift, were also recruited to serve as a comparison group. To prevent potential confounding effects from housework and childcare at home, we enrolled only nurses who were unmarried and not caring for children. The study was conducted during a single season, from March to June 2005 when the average temperature in Taipei ranged between 18°C and 24°C and the duration of daylight ranged between 12 and 13 h, to control for potential seasonal variations of BP and tolerance of shiftwork (McLaughlin et al., 2008; Perez-Lloret et al., 2006; Tsuchihashi et al., 1995).

Subjects were invited to participate voluntarily. They were excluded if they had thyroid dysfunction, diabetes mellitus, hypertension, a history of cardiovascular disease (stroke, coronary arterial disease, or myocardial infraction), body mass index (BMI) >25 kg/m^2^, or were pregnant, current smokers, or taking contraceptive or sleeping pills. Their eligibility was confirmed by reviewing their medical records. All subjects were female between 25 and 35 yrs of age. The mean age of nurses on the regular day shift and rotating shifts were both 27 yrs, with mean heights, mean body weights, and BMIs for the two groups being, respectively, 162.5 cm and 160 cm, 49.5 kg and 54 kg, and 19 kg/m^2^ and 21 kg/m^2^. The characteristics of the regular day shift group and rotating shift group did not differ (rank sum test). One of the eight OPC nurses resigned and another withdrew due to a skin allergy to the electrode paste, leaving six in that group who completed the study. Our protocol followed the ethical guidelines recommended by the journal (Portaluppi et al., 2008) and was approved by the Institutional Review Board of the hospital before the research began. Written informed consent was obtained from all subjects.

### Study Design

Most nurses at the hospital worked three different shifts: day (08:00–16:00 h), evening (16:00–00:00 h), and night (00:00–08:00 h) shifts. Every nurse was required to work one shift for a full month before rotating to a different shift. According to hospital policy, each nurse worked for 4 consecutive days and took the following day off (off-duty day). Cardiac testing took place at least 2 wk after a subject had begun a new shift. Each shiftworking nurse underwent simultaneous and continuous ambulatory blood pressure monitoring (ABPM) and electrocardiogram (ECG) monitoring during the fourth day of a work cycle as well as the following off-duty day. They underwent ABPM and ECG monitoring every month for 3 consecutive months to cover each nurse as she worked the three different shifts. Because not all of the subjects began the study while on the same shift, the order in which each subject worked the day, evening, and night shifts over the 3-month study period was not uniform. However, this quasirandom distribution or order effect was not expected to affect physiological findings. All of the OPC nurses worked the regular day shift and underwent one session of 48-h ABPM and ECG monitoring only, covering the work day and the next off-day. The ABPM and ECG devices were tested 30 min before the monitoring of each participant began; each participant also recorded the actual waking and sleeping durations during the 48-h monitoring period.

### Twenty-Four-Hour Monitoring of Arterial Blood Pressure

The ABPM was accomplished using a noninvasive, automated oscillometric device, Dynapulse 5000A (Pulse Metric, San Diego, CA). An appropriate cuff, 51.5 × 14.3 cm in size, was placed on the participant's left arm (all participants were right-hand dominant). The investigator calibrated the device every month and took five calibrated readings simultaneously using a standard mercury sphygmomanometer when the subjects were fitted with the device. Subjects were requested to hold their left arm in a natural position at heart level whenever a reading was being taken wherever they were and whatever they were doing. The usual default daytime setting extended from 08:00 to 23:00 h and nighttime from 23:00 to 08:00 h the next day. During the daytime, BP measurements were taken every 15 min, and during the nighttime every 30 min.

### Forty-Eight-Hour Ambulatory ECG Monitoring

Continuous 48-h ambulatory ECG monitoring was performed using a three-channel DR180 recorder and analyzed by Holter LX analysis software (NorthEast Monitoring Inc., Maynard, MA, USA). Each device was pretested twice before actual use for continuous 48-h functional recording at a sampling rate of 1000 Hz (1 ms/cycle).

The ECG wave complex (QRS) was classified as normal sinus rhythm, noise, or atrial and ventricular premature beats by comparing the adjacent QRS morphologic features. The normal-to-normal (NN) intervals were deduced from the adjacent normal sinus beats. The NN interval time series were then transferred to a personal computer and postprocessed by a program written in Matlab language (version 5.2; MathWorks Inc., Natick, MA, USA). The missing intervals of the raw NN data were linearly interpolated and resampled at 4 Hz by the Ron-Berger method (Bigger et al., 1992). The HRV analysis was based on each 5-min segment of the NN intervals. Power spectral density was calculated by Welch's averaged periodogram (Welch, 1967). The frequency domain measurements of HRV included the low frequency (LF) band (0.04–0.15 Hz), which is related to both sympathetic and parasympathetic modulation, and the high frequency (HF) band (0.15–0.40 Hz), which is governed almost exclusively by parasympathetic modulation. The ratio of LF to HF power is often used as a metric of sympathetic-parasympathetic balance (Malliani et al., 1991; Rajendra et al., 2006).

### Statistical Analysis

The hourly means of each HRV variable and BP during the off-duty day were considered baseline values for each subject to yield a total of 24 baseline hourly means per variable. These baseline values were subtracted from each 5-min measurement for HRV (and 15–30 measurements for BP) taken within the same hour of any day to obtain the circadian pattern–adjusted measurements for each subject. The circadian pattern–adjusted measurements obtained during the work day were further divided into three time periods: the 8-h working period, the sleeping period, and the wake-time of the nonworking period. The off-duty day was also divided into three corresponding periods: a sleeping period, which is the actual sleeping time for each nurse, and the remaining hours were divided into two periods corresponding to working and nonworking periods of the previous day; the hypothetical “working period” of the off-duty day was the time period corresponding to the working period of the previous work day, had the nurse continued working on that day. Namely, it was 16 h after the end of the working period of the work day and lasted for 8 h if the nurse was awake.

For each HRV or BP variable, we let *Y*_*ijkl*_ be the average of the *i*th subject's circadian pattern-adjusted measurements during the hours in the *j*th period of the *k*th day for the *l*th shift, where *j* = 1, 2, and 3 represented working, nonworking, and sleeping periods, *k* = 1 and 2 represented work day and off-duty day, and *l* = 1, 2, 3, and 4 represented day, evening, night shifts, and OPC, respectively. To study the dynamic shift changes in simultaneous HRV and BP recordings on the work day and the following off-duty day, we applied a linear mixed-effect model to the measurement averages of the 16 shiftwork nurses. Specifically, to examine the HRV and BP changes in the same period from day shift to evening shift as well as from day shift to night shift, we used the following model:

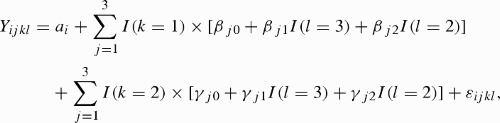
where *a*_*i*_ is a random component normally distributed with mean 0 and *I*(·) is an indicator function and a constant variance. The random error term was correlated among the repeated measurements within each subject. In this model, the fixed parameters *β*_*j*0_, *β*_*j*1_, and *β*_*j*2_ are the overall mean measurement taken on the *j*th period of the work day, mean response difference between night and day shifts, and mean difference between evening and day shifts, respectively. The corresponding three fixed parameters *γ*_*j*0_, *γ*_*j*1_, and *γ*_*j*2_ represent the same values for the off-duty day.

To explore recovery from the effect of working the shifts, we simply examined the deviations of the three periods on the work day and off-duty day of each shift and the outpatient department from the baseline using the following model, which is based on the off-duty day following a day shift:

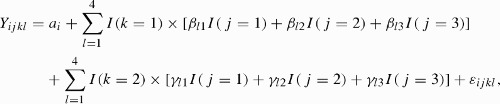



Where *β*_*l*1_, *β*_*l*2_, and *β*_*l*3_ represent the baseline-adjusted mean measurement of working, nonworking, and sleeping periods on the work day for the *l*th shift, respectively. Meanwhile, *γ*_*l*1_, *γ*_*l*2_, and *γ*_*l*3_ are the corresponding three parameters on the off-duty day and are used to measure recovery in 1 day after a *l*th shiftwork. The estimates, standard errors, and *p* values of the model parameters are calculated by the free and popular statistical software R version 2.10.1 (R Development Core Team, 2009) using the function lme for linear mixed-effect model. A *p* value of .05 was considered significant.

## RESULTS

**FIGURE 1:**
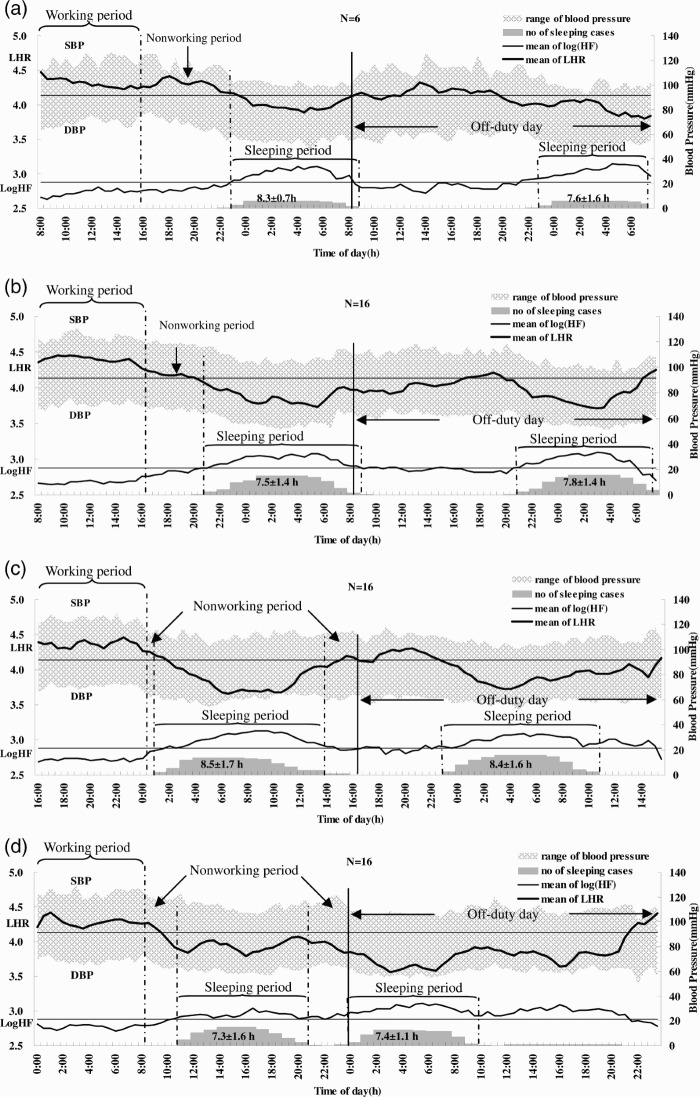
Dynamic changes of simultaneous HRV and BP recordings obtained during a work day and consecutive off-duty day under different shifts. (a) OPC without shift; (b) day shift; (c) evening shift; and (d) night shift. Duration (in hours) of sleeping periods is shown at the bottom of each panel as mean ± SD. The height of the shadow represents the number of subjects who were asleep corresponding to each time period. Two parallel lines indicate mean LHR and Log HF of outpatient clinic nurses (OPC).

The dynamic changes of simultaneous HRV and BP recordings for work days and consecutive off-duty days categorized by shift are summarized in Figure 1, which in the top panel presents the values for the OPC nurses working the regular shift. We found no significant on-duty/off-day or shift differences in sleep duration.

### HRV and Blood Pressure Changes across Each Period of the Work Day and Consecutive Off-Duty Day of Each Shift (Day Shift as a Baseline)

Using a linear mixed-effect model, we found work-time systolic blood pressure (SBP) of the nurses when working the night shift to be significantly higher (9.7 mm Hg) than when working the day shift (*p* < .001). We found the sleep-time DBP of the night shift nurses to be significantly lower than when they were working the day shift. We found no significant differences in LHR and Log HF in the same subjects working night and evening shifts during the three measurement periods on the work compared to their own day-shift values.

The LHRs were significantly lower and the SBPs and DBPs significantly higher on the off-day following night shifts than those following day shifts for the wake-time corresponding to the working period (Table 1). The Log LF and Log HF were significantly higher on the off-day following a night shift than they were following a day shift for the wake-time corresponding to the nonwork period of the previous work day.

**TABLE 1:** Regression coefficients and standard errors (SE) resulting from construction of the linear mixed-effect model for circadian rhythm–adjusted parameters of HRV and BP changes across each period of the work and consecutive off-duty day of each shift (day shift as a baseline)

Period	Shift	MRR	Log LF	Log HF	LHR	SBP	DBP
*Work day*							
Working period	Average	−128.92 (31.7)**	−0.143 (0.034)**	−0.190 (0.043)**	0.419 (0.103)**	11.3 (2.9)**	9.8 (2.0)**
	Night vs. Day	−45.30 (33.3)	−0.034 (0.037)	−0.041 (0.045)	−0.003 (0.103)	9.7 (3.4)**	3.9 (2.2)
	Evening vs. Day	13.25 (32.6)	0.008 (0.036)	0.020 (0.044)	−0.098 (0.100)	4.7 (3.4)	0.3 (2.2)
Nonworking period	Average	16.06 (31.7)	0.016 (0.034)	0.017 (0.043)	−0.018 (0.103)	7.7 (2.9)**	4.4 (1.9)*
	Night vs. Day	−13.30 (33.3)	−0.014 (0.037)	−0.017 (0.045)	0.002 (0.103)	2.9 (3.4)	0.9 (2.2)
	Evening vs. Day	−4.17 (32.6)	0.002 (0.036)	−0.004 (0.044)	0.065 (0.100)	−2.1 (3.4)	−1.1 (2.2)
Sleeping period	Average	42.27 (31.7)	0.042 (0.034)	0.044 (0.043)	−0.047 (0.103)	4.0 (2.9)	1.0 (1.9)
	Night vs. Day	28.94 (33.3)	0.032 (0.037)	0.046 (0.045)	−0.085 (0.103)	−5.2 (3.4)	−5.3 (2.2)*
	Evening vs. Day	49.73 (32.6)	0.061 (0.036)*	0.075 (0.044)	−0.117 (0.100)	−2.5 (3.4)	1.4 (2.2)
*Off-duty day*							
Wake-time = working period	Average	0.4 (29.6)	0.000 (0.035)	0.000 (0.040)	0.000 (0.078)	0.3 (2.3)	0.1 (1.7)
	Night vs. Day	10.7 (41.1)	−0.003 (0.049)	0.024 (0.057)	−0.231 (0.113)*	13.3 (3.3)**	6.4 (2.4)**
	Evening vs. Day	10.2 (38.3)	0.014 (0.046)	0.004 (0.053)	0.089 (0.105)	−0.6 (3.0)	−1.4 (2.2)
Wake-time = nonworking period	Average	−0.6 (29.6)	0.000 (0.035)	−0.001 (0.040)	0.002 (0.078)	−0.0 (2.3)	−0.0 (1.7)
	Night vs. Day	93.6 (39.2)*	0.103 (0.047)*	0.124 (0.054)*	−0.163 (0.106)	−0.3 (3.0)	0.2 (2.2)
	Evening vs. Day	79.3 (38.3)*	0.032 (0.046)	0.049 (0.053)	−0.149 (0.105)	−1.8 (3.0)	−1.2 (2.2)
Sleeping period	Average	9.7 (31.2)	0.007 (0.037)	0.011 (0.042)	−0.015 (0.081)	−0.3 (2.3)	−0.1 (1.7)
	Night vs. Day	41.9 (40.5)	0.043 (0.049)	0.062 (0.056)	−0.225 (0.108)*	−0.5 (3.1)	−0.6 (2.2)
	Evening vs. Day	71.6 (39.6)	0.075 (0.048)	0.094 (0.054)	−0.176 (0.107)	1.2 (3.0)	1.8 (2.2)

*p < .05; **p < .01.

MRR = mean of R-R intervals; Log LF = log transformation of low frequency; Log HF = log transformation of high frequency; LHR = ratio of LF/HF; LF, HF, in absolute values, SBP = systolic blood pressure; DBP = diastolic blood pressure.

### Recovery of HRV and Blood Pressure across Each Period of the Work Day and Consecutive Off-Duty Day Per Shift

**FIGURE 2:**
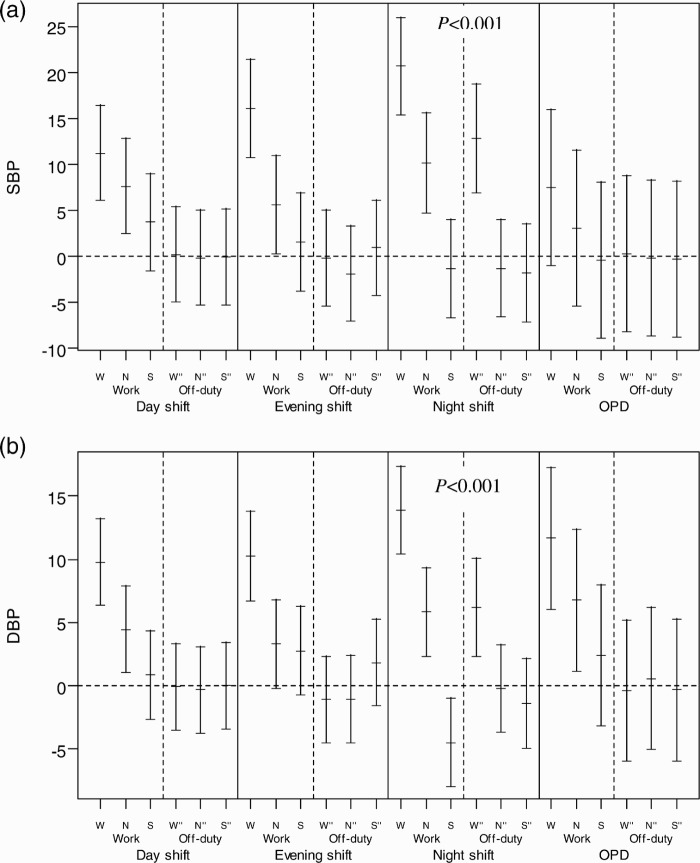
Recovery of HRV and BP across each period of work day and consecutive off-duty day in each shift. The recovery of HRV and BP from different shifts was defined as the BP return to the baseline, which is the hourly mean of the off-duty day after a day shift for each subject. Each HRV and BP measurement was subtracted from the corresponding hourly time-of-day mean to obtain the differences or deviations from baseline. The distributions of all such differences were collected for each period under the three shifts and plotted as the mean and 95% confidence interval. (a) Mean difference of SBP; (b) mean difference of DBP; (c) mean difference of Log HF; (d) mean difference of LHR. W = working period; N = nonworking period; S = sleeping period; W" = time corresponds to working period; N" = time corresponds to nonworking period; S" = sleeping period on the off-duty day.

We found that a subject whose HRV profile changed on the off-duty day after each shift usually returned to the baseline level, but we found that the SBP and DBP of women working the night shift did not completely return to baseline, suggesting incomplete recovery (*p* < .001) (Figure 2). The recovery changes for nurses on a regular day shift (OPC) are also shown in Figure 2 for comparison.

**Figure:**
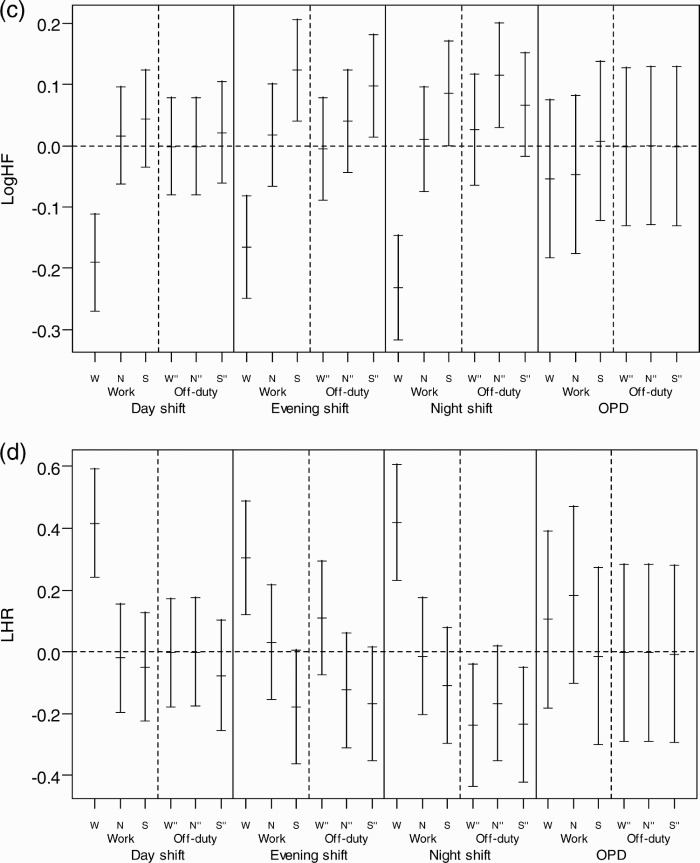


## DISCUSSION

We assessed the repeated-measurements of HRV and BP in the same subject within the same period of time to compare the effects of rotating shifts on HRV and BP and recovery. All participants were of the same race/ethnicity and age range, were normotensive and healthy, did similar work in the same hospital, had the same amount of time to adapt to each shift, and had similar sleeping and respiratory patterns. We subtracted the baseline hourly means for both HRV and BP to obtain the circadian pattern–adjusted measurements for each subject. By conducting a mixed-effect model on these adjusted measurements, we were able to control for individual characteristics to reduce the potential contribution from between-subject variations from genetic predisposition and environmental factors and to explore the effects of shiftwork on the subjects' circadian patterns of HRV, BP, and recovery after different shifts.

In the general population, autonomic activity exhibits a circadian rhythm with predominant sympathetic dominance during the day and relative increase in vagal control during the night. The circadian rhythm in autonomic activity seems to be oscillated by an endogenous clock and synchronized over 24 h by changes in daylight and cyclic changes in daily routine (Turek, 1985). Shiftwork causes a mismatch between the circadian timekeeping system and the work-sleep schedule. As apparent in Table 1, we analyzed HRV (cardiac stress) and BP (vascular stress) changes across each period on a work and off-duty day of each shift by adjusting (or subtracting) the circadian rhythm effect of the day shift. In the present study, the results showed no significant difference in cardiac stress measurements taken during a work day of each shift. The vascular stress of the work-time, in terms of the SBP, when the nurses worked the night shift was 9.7 mm Hg (Table 1), which was higher than when they worked the day shift. The average change of the sleep-period DBP of those working the night shift on a work day was significantly lower (5.3 mm Hg lower) than when they worked the day shift. Previous studies have related night and rotating shiftwork to higher sleep-time SBP compared to day workers (Kario et al., 2002; Yamasaki et al., 1998). These studies, however, did not perform self-comparisons and the data were not adjusted for individual circadian rhythm characteristics. If adjusted for the circadian rhythm effect of day shift for each individual, the DBP of the night shift showed a lower value during the sleeping period of the work day than the day shift.

The changes of SBP and DBP seemed to spike during the waking time, at the same time on the off-day as they did on the work day (Table 1), which indicates a delayed recovery in vascular stress after the night shift. Vascular stress of the night shift appeared to be carried over to the first part of the off-day, namely, the wake-time corresponding to the working period of the previous work day on the off-duty day. But, it did not carry over to the later periods, i.e., the wake-time corresponding to the nonwork period of the previous work day and sleeping periods. The recording of sleeping periods for the night shift found that the average interval between two sleeping periods (those of the work day and consecutive off-duty day) was shorter than those of day and evening shifts, suggesting that the effect of delayed recovery after the night shift could be partially attributed to an interference of the circadian system at the end of such a shift. The above carryover effect might be an early sign of poor circadian adjustment reported by studies of melatonin changes in permanent night-shift workers (Folkard, 2008). In addition, compared with day workers of the outpatient clinic (OPC), shiftworkers showed a significantly higher SBP and LHR and lower Log HF during all shifts (Figure 2), which might suggest increased overall cardiac and vascular stresses in rotating shiftwork nurses than in regular day-work nurses. These results support the association between a greater fluctuation of autonomic modulation over a short period of time and increased frequency of cardiovascular disease in shiftworkers (Åkerstedt et al., 1984; Knutsson et al.,^1986^), and they may also imply that long-term shiftwork might increase the risk of cardiovascular events (Kristensen, 1989).

There was a delay in the recovery of SBP and DBP on the following off-duty day after the night shift (Figure 2). These findings suggest the residual effect of vascular stress of night-shiftwork may carry over to the next off-duty day; this corroborates the findings of previous studies showing such poor recovery as evidenced by hand-ear neuromotor response, reaction time, and cognitive performance (Axelsson et al., 2008; Ishii et al., 2004; Matsumoto et al., 1996; Sallinen et al., 2008; Totterdell et al., 1995). In contrast, the differences of LHR and Log HF significantly improved during the off-duty day for the three corresponding periods (*p* < .001), suggesting recovery from cardiac stress (Figure 2). In an informal interview with participants in this study, almost all complained of fatigue after a night shift and described a better quality of sleep the following off-duty day, as was reflected by significant reduction of LHR on the off-duty day (Table 1). However, we did not collect information on cardiac stress and vascular stress for the second off-duty day. Further research may be needed to determine exactly how many off-duty days in total are needed for complete recovery from the night-shift work.

This study has some limitations. First, we could only recruit a small sample size of subjects to complete the study because of the discomfort related to using the equipment of both ABPM and Holter for 48 h. We also did not record breathing frequency during the 48-h Holter test. However, because we analyzed circadian rhythm-adjusted measurements for each participant and then based our analysis upon repeated-measurements taken from the same person, we assume that the breathing frequency and disturbance of wearing the equipment would be about the same for each individual person. Second, we were unable to conduct more questionnaire investigations to assess the subjective feelings and self-ratings of sleep quality, fatigue, and alertness in quantitative terms during and after each shift, which would have provided a more informative connection with our objective study on autonomic system activity. Third, we only recorded HRV and BP for a single 48-h period because of the inconvenience of wearing two types of monitoring equipment; therefore, we were unable to further study how many days would be needed to achieve complete recovery after the night shift.

In conclusion, night shifts may cause more vascular stress than evening and day shifts, and this stress extends to the following off-duty day.
